# MR-guided microwave ablation of hepatocellular carcinoma (HCC): is general anesthesia more effective than local anesthesia?

**DOI:** 10.1186/s12885-021-08298-2

**Published:** 2021-05-17

**Authors:** Zhaonan Li, Chaoyan Wang, Jing Li, Zaoqu Liu, Dechao Jiao, Xinwei Han

**Affiliations:** Department of Interventional Radiology, First Affiliated Hospital of Zhengzhou University, No. 1 Jianshe East Road, Zhengzhou City, 450000 Henan Province China

**Keywords:** Hepatocellular carcinoma, Microwave ablation, Interventional radiology, Magnetic resonance imaging

## Abstract

**Background:**

Percutaneous magnetic resonance-guided (MR-guided) MWA procedures have traditionally been performed under local anesthesia (LA) and sedation. However, pain control is often difficult to manage, especially in some cases when the tumor is large or in a specific location, such as near the abdominal wall or close to the hepatic dome. This study retrospectively compared the results of general anesthesia (GA) and local anesthesia (LA) for MR-guided microwave ablation (MWA) in patients with hepatocellular carcinoma (HCC ≤ 5.0 cm) to investigate whether different anesthesia methods lead to different clinical outcomes.

**Methods:**

The results of the analysis include procedure-related complications, imaging response, and the time to complete two sets of procedures. According to the type of anesthesia, the Kaplan-Meier method was used to compare the local tumor progression (LTP) of the two groups who underwent MR-guided MWA.

**Results:**

All patients achieved technical success. The mean ablation duration of each patient in the GA group and LA group was remarkably different (*P* = 0.012). Both groups had no difference in complications or LTP (both *P* > 0.05). Notably, the tumor location (challenging locations) and the number of lesions (2–3 lesions) could be the main factors affecting LTP (*p* = 0.000, *p* = 0.015). Univariate Cox proportional hazard regression indicated that using different anesthesia methods (GA and LA) was not associated with longer LTP (*P* = 0.237), while tumor location (challenging locations) and the number of lesions (2–3 lesions) were both related to shorter LTP (*P* = 0.000, *P* = 0.020, respectively). Additionally, multivariate Cox regression further revealed that the tumor location (regular locations) and the number of lesions (single) could independently predict better LTP (*P* = 0.000, *P* = 0.005, respectively).

**Conclusions:**

No correlation was observed between GA and LA for LTP after MR-guided MWA. However, tumors in challenging locations and the number of lesions (2–3 lesions) appear to be the main factors affecting LTP.

## Background

Until a few decades ago, surgical resection was the only effective choice for the treatment of hepatocellular carcinoma (HCC). However, various effective modalities have been shown, including local ablation and liver transplantation [1, 2]. Due to the shortage of donor’s livers and high cost, many patients are not candidates for these radical options. Of note, ablation has been established as the standard treatment for small HCC and has shown the same oncological results as surgical resection in randomized studies [3–5]. Therefore, thermal ablation is widely accepted and applied for early HCC in most centers.

MR-guided thermal ablation has been used in all aspects of tumor treatment and has shown favorable technical feasibility and safety [6–9]. Most MR-guided minimally invasive treatments are performed under local anesthesia (LA). Although the treatment process under LA is more economical and speeds up the procedure and patient recovery, the MR-guided ablation process has a long scanning time and intense noise, greatly affecting the patient’s treatment experience. Furthermore, due to the magnetic field design of the closed-loop, some patients cannot receive the entire procedure in a small space. Therefore, general anesthesia (GA) may be a better choice for MR-guided treatment. GA can improve the comfort of the patient during the procedure and ensure the effective implementation of the entire process. Moreover, studies have shown that the choice of anesthesia may affect the clinical prognosis of patients with malignant tumors [10]. Tumor recurrence involves many causes, and anesthesia methods and anesthetics have recently attracted widespread attention [11, 12].

Currently, no data have been presented that compare the tumor recurrence rate of GA or LA in MR-guided microwave ablation (MWA) treatment. This study explored the relationship between anesthesia techniques (GA and LA) and local tumor progression and attempted to establish a regression model to further determine the effect of GA on tumor prognosis.

## Methods

### Patients

This was a retrospective cohort study conducted in a single center approved by the institutional review board. We included 34 patients (53.4 ± 7.5 years; range 42–67 years) who received GA for MR-guided MWA and 38 patients (52.1 ± 8.5, range 43–69 years) who received LA for MR-guided MWA (Tables 1 and 2). The inclusion and exclusion criteria are listed in Table 3.

**Table 1:** Patient characteristics

Characteristics	GA group (*n* = 34)	LA group (*n* = 38)	*P* value
**Age**	53.4 ± 7.5	52.1 ± 8.5	0.254^‡^
**Sex**			0.149*
Male	24	20	
Female	10	18	
**ECOG performance status**			1.000*
0	27	30	
1	7	8	
**Etiology**			0.487^§^
Hepatitis B	5	3	
Hepatitis C	19	25	
Alcohol	8	6	
Unknown	2	4	
**Child–Pugh class**			1.000^*^
A	20	23	
B	14	15	
**Location of tumour**			1.000*
Challenging locations	17	18	
Other parts	17	20	
**AFP level (ng)**			0.808*
<200	21	25	
>200	13	13	
**Tumour diameter**			1.000*
< 3 cm	18	20	
3⩾cm,< 5 cm	16	18	
**Tumour number**			0.633*
Single(1)	22	25	
2	7	10	
> 3	5	3	
**Puncture score**			0.263*
3–4	24	22	
1–2	10	16	
**Duration of procedure (min)**	128.7 ± 40.3	90.8 ± 33.3	0.003^‡^
**Generator power (wt)**	55.5 ± 5.6	50.7 ± 6.1	0.012^‡^

Note.—Unless indicated, data are numbers of patients, and numbers in parentheses are percentages

*ECOG* Eastern Cooperative Oncology Group. Note—general anesthesia (GA) and local anesthesia (LA); Challenging locations(Hepatic dome, Close to the heart/diaphragm/hepatic hilum)

See Table 2 for details of puncture scoring standards

*Pearson χ2 test was used. §Fisher exact test was used

**Table 2:** Scoring standards for puncture performance

Score	Standards
**0**	Unsuccessful needle insertion
**1**	Successful puncture requiring > 4 needle repositions for single lesion
**2**	Successful puncture requiring 3–4 needle repositions for single lesion
**3**	Successful puncture requiring 1–2 needle repositions for single lesion
**4**	Successful first puncture

**Table 3:** Inclusion and exclusion criteria

Inclusion criteria	*Exclusion criteria*
1 Age range: 18–75 years	Age < 18 or > 75 years
2 HCC diagnosed according to EASL standards	No pathology or image evidence
3 Child–Pugh grade A or B	Child–Pugh grade C
3 BCLC grades are A and B	BCLC grades are C
4 ECOG score≦2	ECOG score > 2
4 Liver lesions ≤3	The liver lesions number > 3
5 Single tumour diameter < 5 cm	Single tumour diameter≧5
6 The expected survival time > 3 months	The expected survival time ≤ 3 months
7 No portal vein thrombus	Portal vein thrombus
8 No extrahepatic metastases	Extrahepatic metastases
9 PLT > 40 × 109/L or PT ≤25 s	PLT ≤ 40 × 109/L or PT > 25 s

*EASL* European Association for the Study of the Liver, *ECOG* Eastern Cooperative Oncology Group, *PLT* platelet, *PT* prothrombin time, HCC small hepatocellular carcinoma

### Anesthesia mode

**Fig. 1:**
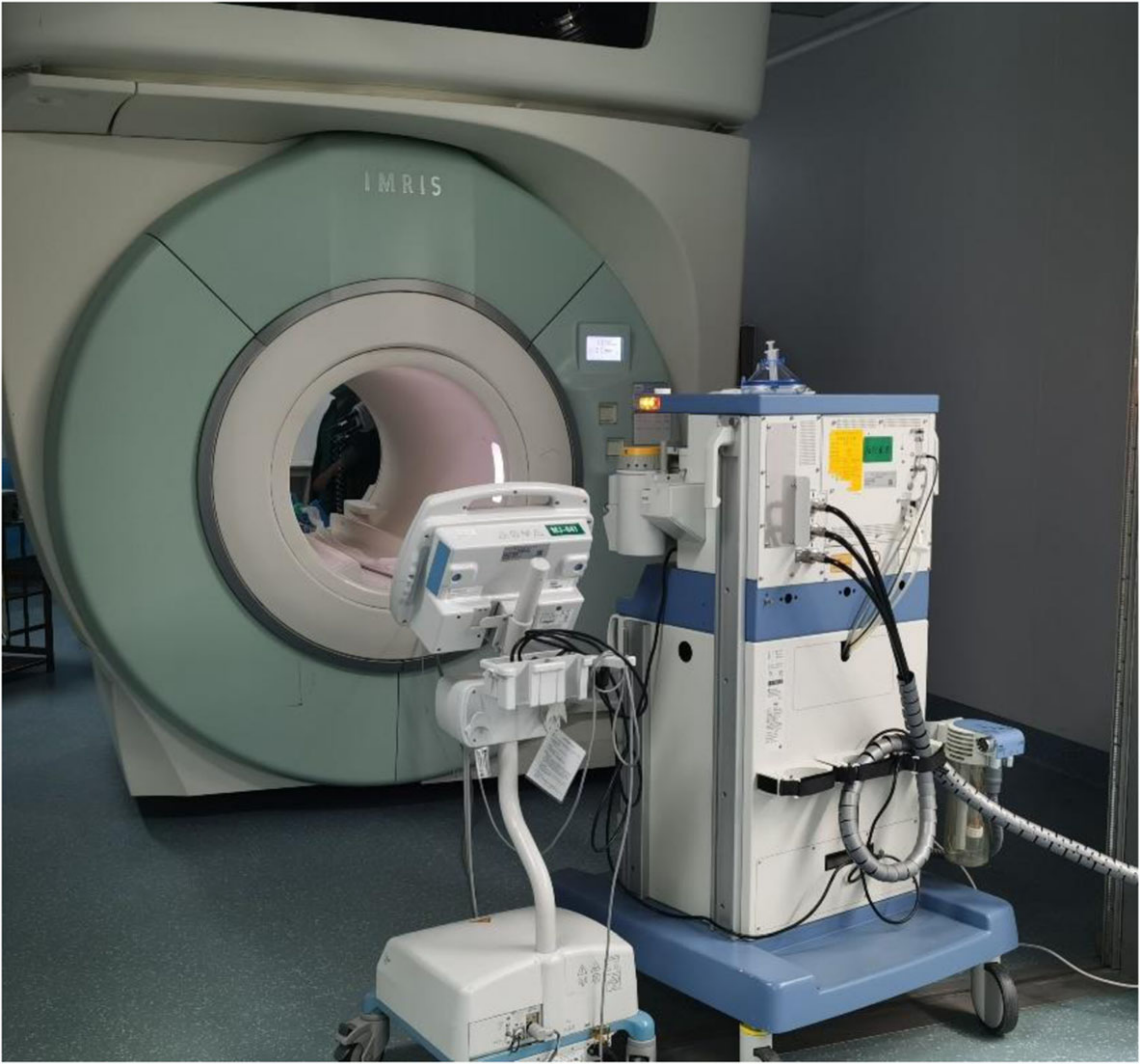
Magnetic resonance imaging and placement of anesthesia equipment during treatment

Patients need sedation, laryngeal mask insertion and hemodynamic monitoring during general anesthesia. The GA group was given propofol, midazolam, and fentanyl/sufentanil to complete anesthesia induction and received laryngeal mask placement and mechanical ventilation. All patients lost consciousness during the procedure. Sevoflurane and/or propofol were used to maintain GA management. The patient recovered in the postanaesthesia care unit (PACU) and was treated with sufentanil or nonsteroidal anti-inflammatory drugs (NSAIDs) for postoperative pain. In the LA group, less than 10 mL of 2% lidocaine was injected subcutaneously at the puncture point. When the patient experienced unbearable pain during the treatment process, NSAIDs could be used to complete intraoperative pain management. The patient was awake and breathed spontaneously during the operation. The placement of MR and anesthesia equipment during treatment is shown in Fig. 1.

### Procedure

**Fig. 2:**
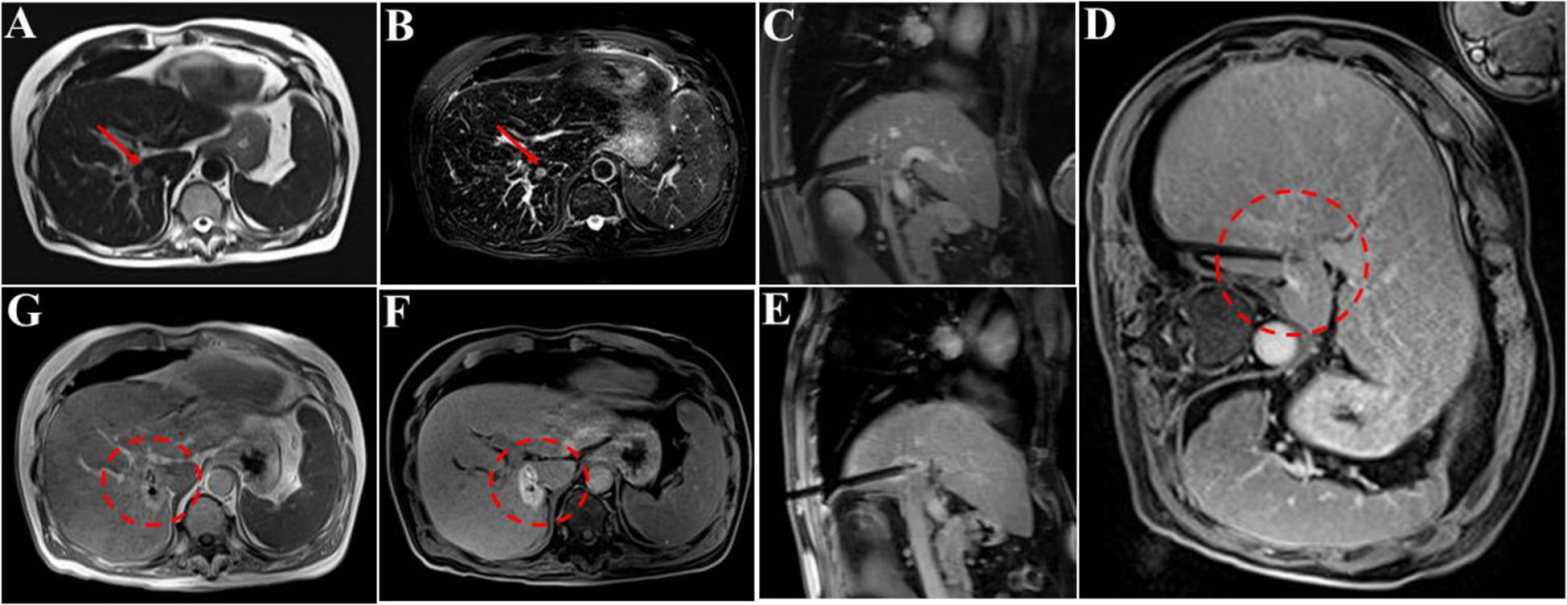
A patient completed MR-guided MWA treatment under general anesthesia. **a-b** The liver caudate lobe (red arrow) has a diameter of 17 mm and appears as hyper-intensity on T2-weighted transverse images before MWA. **c-e** The trajectory of the tilting puncture were adjusted gradually for the lesion target under the guidance of T1WI and the image shows precise insertion of the antenna into the target. **f** After 1 day of MWA, magnetic resonance reexamination found the treatment border covered the lesion completely as hyperintensity on T1WI (dashed circle). **g** Follow-up for 1 month, the lesion (red arrow) was completely ablated

The ablation path was determined according to the preoperative CT / MR. All procedures were performed alternately by two interventional radiologists with 6–10 years of experience in ablation. Routine electrocardiogram and oxygen saturation monitoring (Invivo, Orlando, USA) and MR-compatible MWA apparatus (2450 MHz, ECO Medical Instrument Co., Ltd. Nanjing, China) were placed at a distance of 2.5–3 m beside the MR compatible operating table. After using the cod liver oil capsule matrix to mark the surface of the body, a standard MR protocol was completed to locate intrahepatic lesions. Then, insert the microwave probe (ECO-100AI13, 1.8 mm, 15 cm, Co., Ltd. Nanjing, China) into the center of the tumor under the guidance of MR, and perform multiple scans to confirm that the tip of the applicator was 0.5–1 cm beyond the distal tumor. Besides, the tumor at each site was ablated with 45-65wt for 4–9 min (Fig. 2). During the ablation process, a series of T2 Haste and T1 Vibe sequences were continuously performed every 16 s to monitor the ablated range. Of note, the ablation area needs to reach 110% of the lesion. If the requirements did not satisfy, the probe should be repositioned, and multiple overlapping ablations were needed. The MR scan sequence and parameters used in our study are shown in Table 4.

**Table 4:** MR scanning sequences and parameters

Section	*Sequence*	*TE (ms)*	*TR (ms)*	*Slice thicknes (mm)*	*Matrix*	*Flip angle*	*Band Width (Hz/pixel)*
**Transverse section**	T1 Vive	1.93	4.56	3.3	216 × 288	9.0	400
**Transverse section**	T2 Haste	106	1000	4.5	137 × 256	180	781
**Transverse section**	Diffusion	83	7100	5.0	192 × 144	90	1670
**Coronal section**	T1 vibe	2.46	6.11	3.0	179 × 256	9.0	410
**Sagittal**	T2 Haste	106	1000	4.0	137 × 256	180	781

### Definitions

Local tumor progression is defined as the appearance of tumor foci at the edge of the ablation zone after at least one contrast-enhanced follow-up study has documented adequate ablation and an absence of viable tissue in the target tumor and surrounding ablation margin by using imaging criteria. Tumor in challenging locations means the tumor is close to the hepatic dome/heart/diaphragm/hepatic hilum. Tumor in regular location means the tumor is located in a non-challenging part of the liver. The ablation evaluation standards were based on the modified response evaluation criteria in solid tumors RECIST guidelines (version 1.1) [13]. Intervention-related complications were jointly evaluated according to the National Cancer Institute Common Terminology Criteria for Adverse Events (CTCAE Version 4.03) [14] and Society of Interventional Radiology (SIR) classification system [15].

### Follow up

Electronic medical records were reviewed to collect pre-and post-treatment laboratory results and information on treatment-related complications. Imaging follow-up was performed with contrast-enhanced MR or CT at intervals of 1, 3, and 6 months with a 6-month interval for follow-up thereafter. All post-procedure and follow-up images were reviewed for consensus between a senior radiology resident and a board-certified interventional radiology faculty member with 5 years of experience in oncologic imaging and interventions. If tumor recurrence was found during the period, a second TACE-MWA or MWA procedure was performed separately depending on the patient’s condition. Patients who died early or lost to follow-up were excluded, and each patient met the follow-up time of 36 months.

### Statistical analysis

Statistical software SPSS 22.0 was used (SPSS Inc., Chicago, IL, USA), *P* < 0.05 was considered to indicate a significant difference. To determine the significant difference between the two groups, continuity correction, independent sample t-test, Pearson χ2 test and Fisher exact test were used. Categorical variables are represented as numbers or percentages (%), and continuous variables are represented as means ± Standard deviation (SD). Chi-square test or Wilcoxon rank-sum test was used to compare the two groups. Kaplan-Meier survival curves were used for survival analysis. Univariate and multivariate Cox proportional hazards regression were used to predict the prognostic factors of LTP.

## Result

### Patient characteristics

A total of 72 patients with HCC ≤ 5.0 cm were included in the present study (GA-group, *n* = 34; LA-group, *n* = 38). There were no significant differences in age, gender, ECOG score, etiology, Child-Pugh classification, tumor location, or tumor diameter between the two types of anesthesia. Patients in the GA-group and the LA-group, the mean energy of each tumor was 55.5 ± 5.6wt and 50.7 ± 6.1wt, respectively. The mean ablation duration of each patient in the GA-group and LA-group were 128.7 ± 40.3 and 90.8 ± 33.3, respectively, and the difference between the two groups was statistically significant (*P* = 0.012).

### Safety and complication

Generally, the adverse events and complications were CTCAE Grade 1/2 or Society of Interventional Radiology Grade A/B (Table 5). Specifically, fever (with/without treatment) and postoperative pain were the most common adverse events. Of the four exceptions, the incidences of asymptomatic perihepatic fluid, liver abscess, pleural effusion and subcapsular hepatic hemorrhage in the GA group were 1 (3%), 1 (3%), 2 (6%) and 1 (3%), respectively. In the LA group, the complication rates were 1 (3%), 2 (5%), 1 (3%) and 1 (3%), respectively. It is worth noting that there were no significant differences between the two anesthesia methods. Also, patients with long-term liver abscesses need to be treated with antibiotics and abscess puncture drainage treatment, patients with a large number of pleural effusions were treated with thoracic drainage, and patients with severe subhepatic hemorrhage were treated by interventional embolization in time. All patients had no life-threatening complications during treatment.

**Table 5:** Adverse events and complications

Categories	GA group (***n*** = 34)		LA group (***n*** = 38)		*P*
	Grades				
**Adverse events**	**CTCAE**		**CTCAE**		
Fever, maximum 38 °C, no treatment	I	4 (12)	I	6 (16)	0.740^*^
Fever, > 38 °C,treatment	II	22 (65)	II	25 (66)	1.000^*^
Nausea or vomiting	II	5 (15)	II	3 (8)	0.463^§^
Mild pain, requiring nonopioid oral analgesic treatment	II	17 (50)	II	13 (34)	0.233^*^
Moderate pain, requiring opioid oral analgesic treatment	II	10 (29)	II	9 (24)	0.604^*^
Mild liver dysfunction, requiring conservative treatment	II	12 (35)	II	9 (24)	0.310^*^
**complications**					
Asymptomatic perihepatic fluid	IV	1 (3)	IV	1 (3)	1.000^§^
Liver abscess	III	1 (3)	III	2 (5)	1.000^§^
pleural effusion	III	2 (6)	III	1 (3)	0.599^§^
Subcapsular liver hemorrhage	IV	1 (3)	IV	1 (3)	1.000^§^

National Cancer Institute Common Terminology Criteria for Adverse Event (CTCAE version 4.03)

Society of Interventional Radiology (SIR) classification system for Complications. Data are numbers of events. Data in parentheses are percentages

Note—general anesthesia (GA) and local anesthesia (LA). Data are numbers of events. Data in parentheses are percentages

*Pearson χ2 test was used. §Fisher exact test was used

### Local tumor progression

**Fig. 3:**
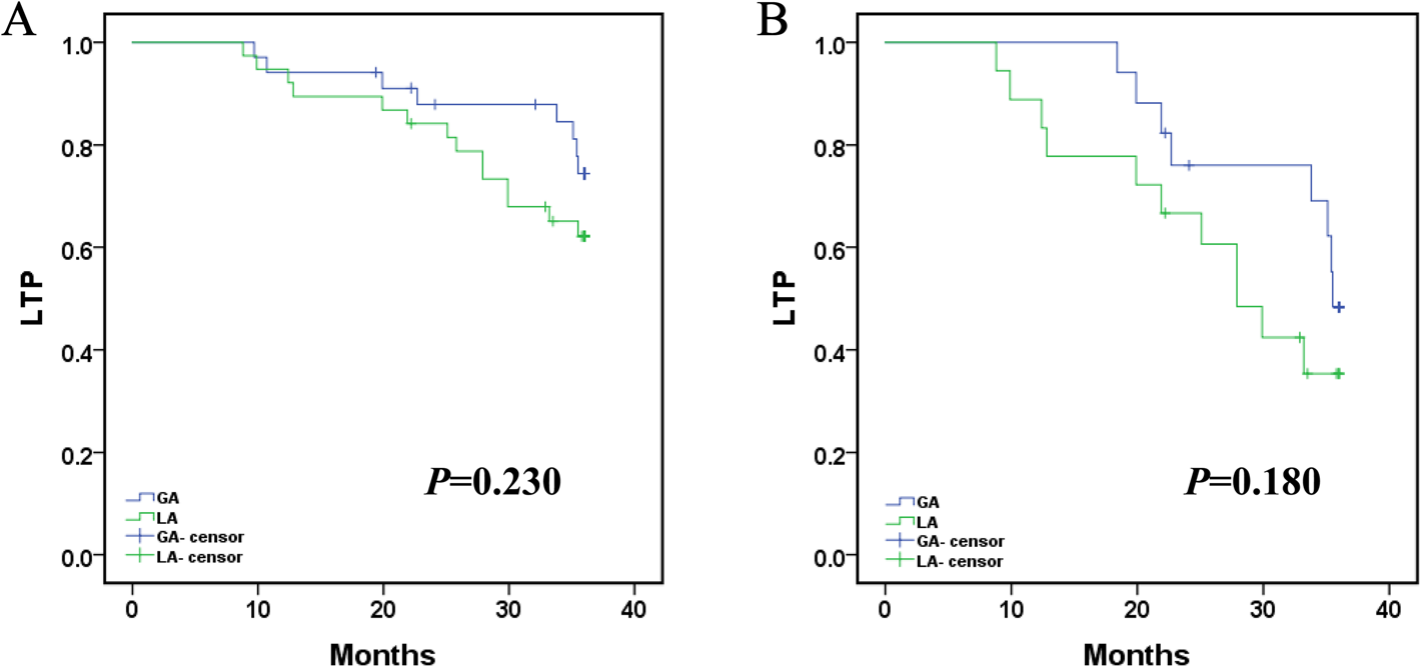
**a**. Comparison of local tumor progression (LTP) after ablation under local anesthesia (LA) and general anesthesia (GA). The mean LTP was 33.434 months (95% CI: 31.133, 35.734) in GA versus 31.132 months (95% CI: 28.535, 33.730) in LA (*p* = 0.230, log-rank test). The 12-, 24-, and 36-month LTP rates in GA were 94.1, 87.9 and 74.4%, respectively, and the 12-, 24-, and 36-month LTP rates in LA were 94.7,84.2 and 62.1%, respectively. **b**. Comparison of different anesthesia methods on LTP of tumor in challenging locations. The mean LTP was 32.055 months (95% CI: 28.973, 35.138) in GA versus 26.551 months (95% CI: 22.049, 31.053) in LA (*p* = 0.180, log-rank test). The 12-, 24-, and 36-month LTP rates in GA were 100.0, 76.0 and 48.4%, respectively, and the 12-, 24-, and 36-month LTP rates in LA were 88.9,66.7 and 35.4%, respectively.(Note: challenging locations--Hepatic dome, close to the heart/diaphragm/hepatic hilum)

Local tumor progression (LTP) after MWA under local anesthesia (LA) and general anesthesia (GA) was compared. The mean LTP was 33.434 months (95% CI: 31.133, 35.734) in GA versus 31.132 months (95% CI: 28.535, 33.730) in LA (*p* = 0.230, log-rank test). The 12-, 24-, and 36-month LTP rates in GA were 94.1, 87.9 and 74.4%, respectively, and the 12-, 24-, and 36-month LTP rates in LA were 94.7, 84.2 and 62.1%, respectively (Fig. 3a). The different anesthesia methods on LTP of tumors in challenging locations were compared. The mean LTP was 32.055 months (95% CI: 28.973, 35.138) in GA versus 26.551 months (95% CI: 22.049, 31.053) in LA (*p* = 0.180, log-rank test). The 12-, 24-, and 36-month LTP rates in GA were 100.0, 76.0 and 48.4%, respectively, and the 12-, 24-, and 36-month LTP rates in LA were 88.9, 66.7 and 35.4%, respectively (Fig. 3b).

### Factors affecting LTP

**Fig. 4:**
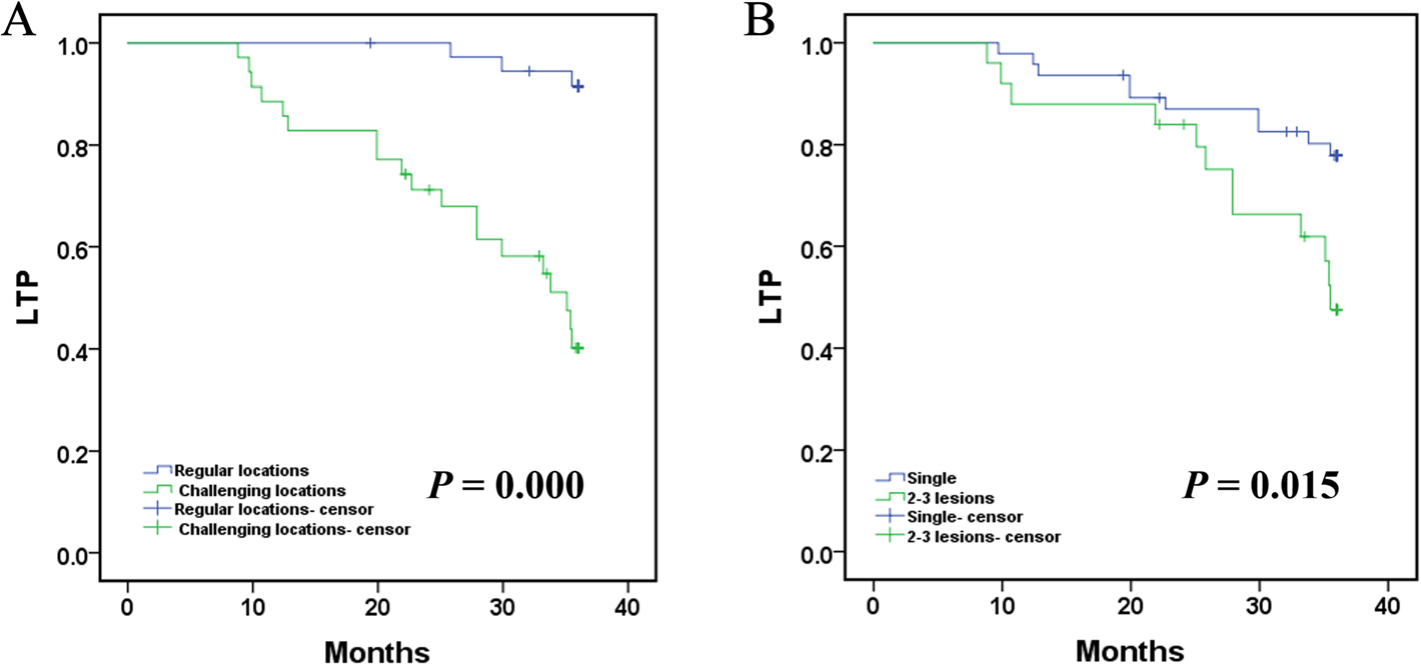
**a**. Comparison of local tumor progression (LTP) between tumor in regular locations and challenging locations after MR-guided MWA treatment. The mean LTP was 35.533 months (95% CI:34.903, 36.162) in regular locations versus 28.607 months (95% CI: 25.423, 31.792) in challenging locations (*p* = 0.000, log-rank test). The 12-, 24-, and 36-month LTP rates in tumor with regular locations were 100.0, 100.0 and 91.6%, respectively, and the12-, 24-, and 36-month LTP rates in tumor with challenging locations were 88.6,71.2 and 40.2%, respectively; **b**. Comparison of local tumor progression (LTP) between single tumors and multiple tumors (2–3 lesions) after MR-guided MWA treatment. The mean LTP was 33.111 months (95% CI: 31.147, 35.075) for procedures with a single lesion versus 30.424 months (95% CI: 26.992, 33.855) for procedures with 2–3 lesions (*p* = 0.015, log-rank test). The 12-, 24-, and 36-month LTP-free survival rates in patients with a single lesion were 97.9, 87.0 and 77.8%, respectively, and the 12-, 24-, and 36-month LTP-free survival rates in patients with 2–3 lesions were 88.0,84.0 and 47.6%, respectively. (Note: challenging locations--Hepatic dome, close to the heart/diaphragm/hepatic hilum)

Univariate Cox proportional hazard regression indicated that using different anesthesia methods (GA and LA) was not associated with longer LTP (*P* = 0.237), while tumor location (challenging locations) and the number of lesions (2–3 lesions) were all related to shorter LTP (*P* = 0.000 and *P* = 0.020, respectively). Additionally, multivariate Cox regression further revealed that the tumor location (regular locations) and the number of lesions (single) could independently predict better LTP (both *P* < 0.005) (Table 6). More specifically, the mean LTP was 35.533 months (95% CI: 34.903, 36.162) in regular locations versus 28.607 months (95% CI: 25.423, 31.792) in challenging locations (*p* = 0.000, log-rank test). The 12-, 24-, and 36-month LTP rates in tumors with regular locations were 100.0, 100.0 and 91.6%, respectively, and the 12-, 24-, and 36-month LTP rates in tumors with challenging locations were 88.6, 71.2 and 40.2%, respectively (Fig. 4a). The mean LTP was 33.111 months (95% CI: 31.147, 35.075) for procedures with a single lesion versus 30.424 months (95% CI: 26.992, 33.855) for procedures with 2–3 lesions (*p* = 0.015, log-rank test). The 12-, 24-, and 36-month LTP-free survival rates in patients with a single lesion were 97.9, 87.0 and 77.8%, respectively, and the 12-, 24-, and 36-month LTP-free survival rates in patients with 2–3 lesions were 88.0, 84.0 and 47.6%, respectively (Fig. 4b).

**Table 6:** Factors affecting LTP

Parameters	LTP			***P***	LTP			***P***
	HR	*95%*CI			*HR*	*95%CI*		
		Univariate Cox’s regression				Multivariate Cox’s regression		
		Lower	Higher			Lower	Higher	
Age (> 60 vs ≤ 60)	0.811	0.330	1.989	0.647	.862	0.336	2.209	0.757
AFP (> 200 vs ≤ 200 ng/mL)	1.422	0.614	3.295	0.411	2.230	0.912	5.450	0.079
Tumour diameter (3⩾,< 5 vs < 3 cm)	0.783	0.334	1.832	0.572	2.766	0.787	9.722	0.113
Tumour location (challenging locations vs regular locations)	35.832	10.530	3.095	**0.000**	27.843	5.718	135.571	**0.000**
Number of lesion (single VS 2–3 lesions)	2.712	1.169	6.294	**0.020**	4.615	1.571	13.556	**0.005**
Child–Pugh stage (A vs B)	1.456	0.629	3.373	0.380	1.668	0.539	5.156	0.375
Anesthesia (GA VS LA)	1.690	0.709	4.032	0.237	2.465	1.003	6.061	0.049

Note—general anesthesia (GA) and local anesthesia (LA); Challenging locations(Hepatic dome, Close to the heart/diaphragm/hepatic hilum)

## Discussion

Radiofrequency ablation (RFA) and microwave ablation (MWA) are the most commonly used as alternative therapeutic options for HCC. Although the technical characteristics of RFA and MWA are quite similar, there are differences in the thermogenesis mechanisms of the two therapies. During the RFA procedure, the heat is restricted to zones of high current density, while during the MWA, it is produced in the fixed space around the microwave probe. The low energy density in the ablative zone under RFA conditions cannot reach the thermally toxic temperature in the nodules close to the cooling vasculature. Fortunately, MWA can bring the target lesion to a higher temperature in a shorter period and produce a larger ablation zone, thereby reducing the heat sink effect on the MWA treatment result [[16, 17].

Currently, most institutions perform percutaneous MR-guided MWA procedures under local anesthesia (LA) and sedation. However, pain control has always been an important issue in the ablation process. When the tumor is adjacent to the abdominal wall or near the hepatic dome, the pain caused by the ablation is more severe than other tumors in regular locations. If intraoperative pain cannot be well controlled, interventional procedures will undoubtedly be affected. Besides, the use of low-efficacy LA is suboptimal for intense intraoperative pain management, which may affect the patient’s respiratory activity and lead to respiratory depression or respiratory arrest. Moreover, due to the claustrophobic environment in the MRI room and the intense noise generated during the procedures, some patients could experience severe anxiety and insecurity and cannot complete the treatment process, resulting in the insufficient tumor ablation area. Notably, there may be a remarkable difference in the rate of local recurrence between patients who have reached a sufficient ablation area and those who have not. Therefore, pain management in MR-guided MWA is a critical condition to ensure adequate tumor ablation. As the preferred alternative to LA, general anesthesia (GA) can produce deeper sedation and better analgesia for patients undergoing MWA procedures in the MRI room, thereby ensuring efficient and safe completion of ablation treatment. In the course of this study, we found that intravenous GA could ensure more stable hemodynamics, but there was no significant difference in postoperative complications between the two anesthesia regimens.

Some studies have shown that different anesthesia may affect the long-term results of cancer treatment [18, 19]. The retrospective analysis of Lai et al. [20] demonstrated that treatment of small HCC with RFA under GA is associated with reducing the risk of cancer recurrence. Moreover, the study by Wang et al. further revealed that the use of GA in the management of thermal ablation and anesthesia could significantly improve the survival time of patients [21]. In these studies, GA may have had a small and temporary effect on the suppression of NK cell cytotoxicity (NKCC) [22]. MWA under LA is often painful because of referred pain, which may force the physician to decrease the current intensity, shorten the coagulation duration, or limit the number of overlapping coagulations [23]. Additionally, another advantage of GA is that systolic blood pressure can be reduced, with the goal of decreasing hepatic blood flow and increasing coagulation diameter [24]. All of the above may account for these results. However, in this research, the difference in anesthesia did not seem to have an impact on the LTP rate of patients (*P* = 0.230). Moreover, we conducted a stratified study on the LTP of HCC in challenging sites and reached a similar conclusion (*P* = 0.180). Multivariate Cox regression revealed that the tumor location (regular locations) and the number of lesions (single) could independently predict better LTP (*P* < 0.005). Therefore, during the limited follow-up period, MR-guided MWA under different anesthesia methods did not seem to have a significant effect on LTP.

There are several shortcomings of our research. Due to software limitations, this study did not use the real-time MR thermometry technique. Second, the cost of MRI-guided ablation treatment under general anesthesia is considerably higher than that of local anesthesia, making it inaccessible for certain patients. Third, although there were fewer local complications of the ablation process in patients under GA, the rate of LTP between the two groups was equivalent, which should be an acceptable result. This study could provide a reference for the selection of anesthesia methods for ablation therapy under the guidance of magnetic resonance in the future. Finally, this is a single-center retrospective control study relating to a small number of cases. In the future, this study needs to be combined with a prospective multicenter study, extend the follow-up time, and increase the overall survival data to reduce the risk of bias.

## Conclusions

Different anesthesia methods seem to have no significant effect on treatment-related complications and LTP in HCC (≤5.0 cm). Secondly, the number of lesions (Single) and tumor location (regular locations) may be associated with favorable LTP. However, Prospective trials exploring the effects of different anesthetic methods on cancer outcomes in these patients are warranted.

## Data Availability

The datasets generated for this study are available on request to the corresponding author.

## Abbreviations

LA: Local anesthesia
GA: General anesthesia
MR: Magnetic resonance
CT: Computed tomography
MWA: Microwave ablation
HCC: Hepatocellular carcinoma
PACU: Post-Anaesthesia Care Unit
NSAIDs: Non-steroidal antiinflammatory drugs
EASL: European Association for the Study of the Liver
AASLD: American Association for the Study of Liver Diseases
LTP: Local tumor progression
RECIST: Modified response evaluation criteria in solid tumours
CTCAE: National Cancer Institute Common Terminology Criteria for Adverse Events
SIR: Society of Interventional Radiology
RFA: Radiofrequency ablation
CA: Cryoablation
NKCC: NK cell cytotoxicity
AFP: Alpha-fetoprotein
